# Characteristics of the portal vein thrombosis recurrence pattern without liver parenchymal invasion from colorectal cancer: a case report

**DOI:** 10.1186/s40792-018-0518-0

**Published:** 2018-09-04

**Authors:** Tetsuya Mochizuki, Tomoyuki Abe, Hironobu Amano, Kenji Nishida, Takuya Yano, Hiroshi Okuda, Tsuyoshi Kobayashi, Hideki Ohdan, Shuji Yonehara, Toshio Noriyuki, Masahiro Nakahara

**Affiliations:** 1 Department of Surgery, Onomichi General Hospital, Onomichi, Hiroshima Japan; 2 Department of Pathology, Onomichi General Hospital, Onomichi, Hiroshima Japan; 3 Department of Gastroenterological and Transplant Surgery, Graduate School of Biomedical and Health Sciences, Hiroshima University, Hiroshima, Japan; 4 Department of Pathology, Okayama University Graduate School of Medicine, Dentistry and Pharmaceutical Sciences, Okayama, Japan

**Keywords:** Rectal cancer, Tumor thrombosis, Surgical resection

## Abstract

**Background:**

Portal vein tumor thrombosis from colorectal cancer is rare, and this recurrence pattern was mainly reported in patients with renal cell carcinoma and hepatocellular carcinoma. Furthermore, the recurrence pattern of portal vein tumor thrombosis without liver parenchymal invasion from colorectal carcinoma has not been previously reported. Herein, we present a patient with progressive portal vein tumor thrombosis without liver parenchymal invasion following curative resection.

**Case presentation:**

A 61-year-old man with a chief complaint of constipation with abdominal pain associated with rectal carcinoma was admitted to our hospital. Computed tomography (CT) showed that the rectosigmoid colon wall was thickened, regional lymph nodes were swollen, and the light space-occupying lesion (SOL) was detected at segment 8 (S8). Neoadjuvant chemotherapy was performed, which was followed by laparoscopic anterior resection. The final diagnosis was stage IIIb (SS, N2, M0). After operation, systemic adjuvant chemotherapy was introduced. At first, tumor marker levels were within the normal range and there were no accumulations on positron emission tomography (PET). Tumor marker levels were elevated, and contrast-enhanced CT demonstrated that the portal vein SOL slowly extended from S8 to S5. Additionally, PET showed that the standardized uptake value was abnormally high at 5.8. Based on the diagnosis of portal vein tumor thrombosis, right hepatectomy was performed. On pathological analysis, tumor thrombosis was associated with rectal carcinoma, and there was no invasion toward the liver parenchyma. Additionally, the surgical cut end was tumor free. Six months after the hepatectomy, the paraaortic lymph nodes showed swelling. The patient is currently undergoing systemic chemotherapy.

**Conclusion:**

Aggressive surgical resection should be considered in cases of portal vein tumor thrombosis. A good long-term prognosis could be obtained by a combination of curative resection and systemic chemotherapy.

## Background

Hepatectomy for resectable liver metastasis from colorectal cancer is the gold standard treatment approach [1–3]. The risk factors for poor prognosis after hepatectomy include a short interval between primary surgery and recurrence, elevated tumor marker levels, multiple tumors, presence of lymph node metastasis, and a large tumor size [3, 4]. However, the efficacy of perioperative systemic chemotherapy has not been established. Regardless of curative surgery, the 5-year recurrence rate after hepatectomy has been reported to be 36–58% [5–7].

Considering the hypothesis that colorectal cancer tumor metastasis progresses from liver parenchyma through the portal vein wall, circulating tumor cells (CTCs) are believed to play an important role [8, 9]. To our knowledge, the recurrence pattern of portal vein tumor thrombosis (PVTT) without liver parenchymal invasion from colorectal carcinoma has not been previously reported. Herein, we present a patient with progressive PVTT without liver parenchymal invasion following curative resection.

## Case presentation

We explained the case report and publication process to the patient and obtained his permission to publish this report.

**Fig. 1:**
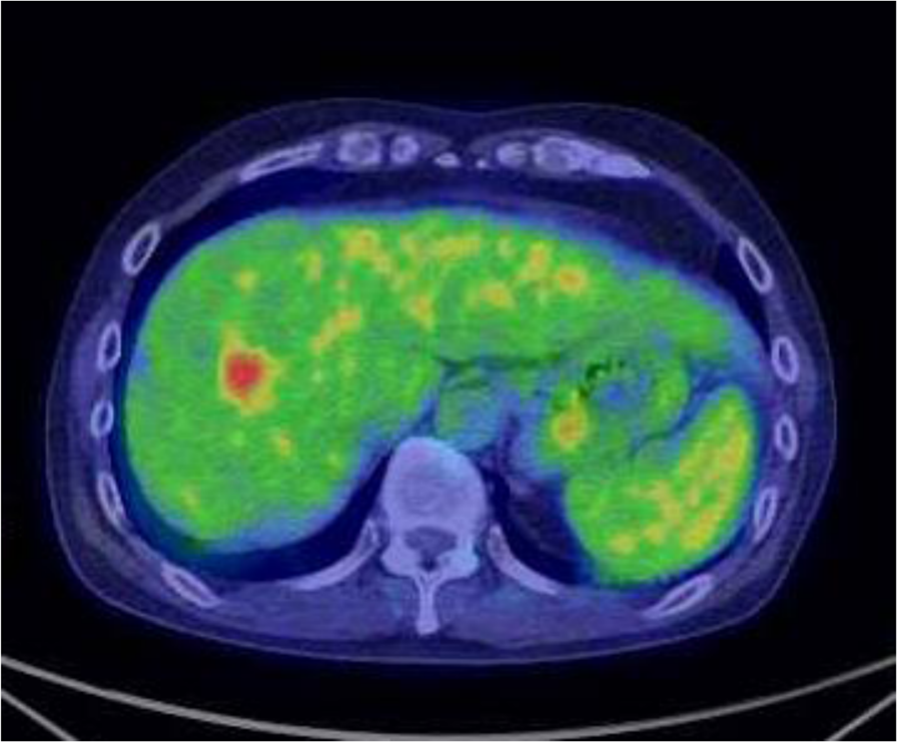
Positron emission tomography-computed tomography after six courses of adjuvant chemotherapy. Imaging showed abnormal accumulation (maximum standardized uptake value of 5.8) in segment 8

**Fig. 2:**
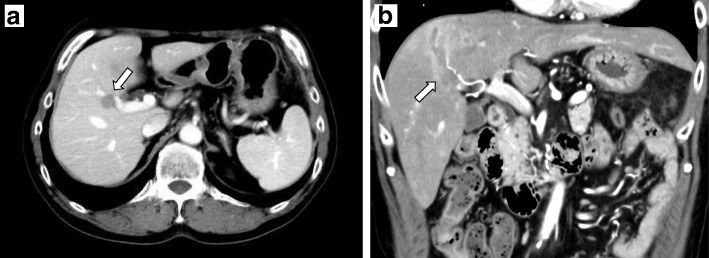
Enhanced abdominal computed tomography. **a** Imaging showed portal vein thrombosis in P8 (white arrow). **b** The tumor thrombosis was extended from P8 to the root of P5, and slight inflammation surrounding the portal vein was detected (white arrow)

**Fig. 3:**
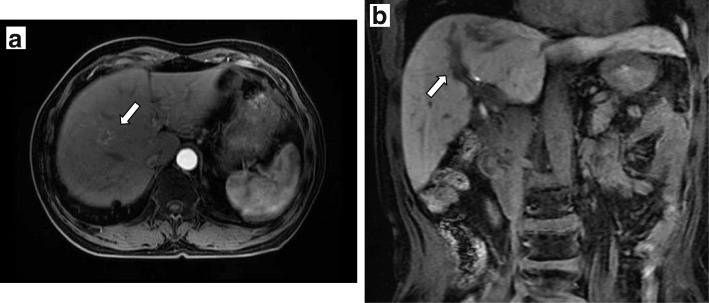
Magnetic resonance imaging with gadolinium ethoxybenzyl diethylenetriaminepentaacetic acid. **a** Imaging detected a ring-like space-occupying lesion within P8 on dynamic study. **b** The tumor thrombosis progressed at the roots of the P5 and P8 branches

**Fig. 4:**
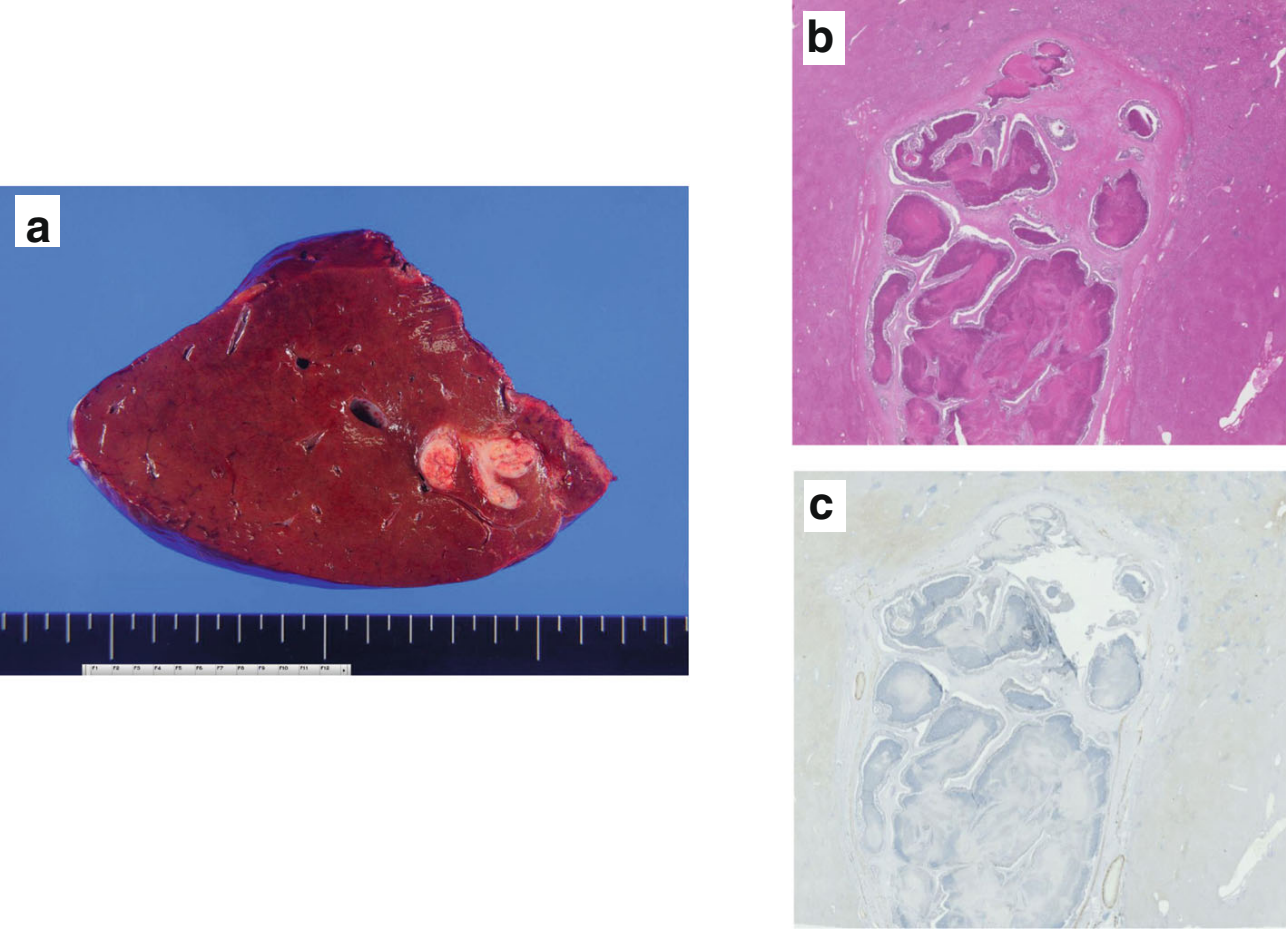
Images of the resected specimen. **a** The tumor did not invade the hepatic parenchyma. **b** Venous infiltration was found in the specimen on hematoxylin and eosin staining (loupe). **c** Tumor thrombosis did not invade the liver parenchyma on desmin staining (loupe)

A 61-year-old man was examined by a local physician for a chief complaint of constipation with abdominal pain. Computed tomography (CT) showed that the rectosigmoid colon wall was thickened, regional lymph nodes were swollen, and the obscure space-occupying lesion (SOL) was detected at S8, especially localized into the portal vein. He was admitted to our hospital for further treatment. Colonography revealed a type 3 tumor in the rectosigmoid colon. Laboratory data demonstrated elevated tumor marker levels (carcinoembryonic antigen, 74.4 ng/mL; cancer antigen 19-9, 53.5 U/mL). Because of obstructive colitis that was associated with his massive cancer, emergency colonostomy was performed. Prior treatment with systemic chemotherapy was performed for curative surgery with suspicion of PVTT: 6 courses of mFOLFOX6 + panitumumab chemotherapy (panitumumab was administered as a 60-min intravenous infusion before oxaliplatine at a dose of 6 mg/kg, leucovorin at 200 mg/m^2^, oxaliplatin at 85 mg/m^2^, and bolus fluorouracil at 400 mg/m^2^, all on day 1, followed by 2400 mg/m^2^/46 h, each 14-day cycle) were administered. Six months after admission, laparoscopic anterior resection was performed. On pathological assessment, the tumor was classified as a moderately differentiated adenocarcinoma (Rs, type 2, 60 × 40 mm in size, whole-circumferential growth, SS, P0, H0, M[−], ly1, v1, N2, D2, aw[−], ow[−], ew[−], CurA), and the final pathological stage was IIIb. Six courses of mFOLFOX6 chemotherapy (leucovorin at 200 mg/m^2^, oxaliplatin at 85 mg/m^2^, and bolus fluorouracil at 400 mg/m^2^, all on day 1, followed by 2400 mg/m^2^/46 h, each 14-day cycle) were administered as adjuvant chemotherapy, during which tumor marker levels were elevated. On positron emission tomography (PET), abnormal accumulation (maximum standardized uptake value [SUVmax], 5.8) at P8 was detected (Fig. 1). CT showed low intensity in the portal vein (Fig. 2a, b). Magnetic resonance imaging (MRI) with gadolinium ethoxybenzyl diethylenetriaminepentaacetic acid revealed that the nodule in the portal vein extended from segment 8 (S8) to S5 and had a ring-like high contrast (Fig. 3a, b). Therefore, right hemihepatectomy was performed (operation time, 364 min; bleeding volume, 300 mL). On histopathological analysis, the PVTT was from colon cancer, which had not invaded the hepatic parenchyma. The cut surface was free from tumor invasion (Fig. 4). The patient had no specific postoperative complications, and he was discharged 13 days after the operation. Four months after hepatectomy, paraaortic lymph node recurrence occurred. The patient is currently undergoing systemic chemotherapy.

### Discussion

Venous tumor thrombosis occasionally occurs in patients with renal cell carcinoma, pancreatic carcinoma, gastric carcinoma, hepatocellular carcinoma (HCC), adrenal cortical carcinoma, and testicular carcinoma [10–14]. To the best of our knowledge, it is rare for a case to exhibit portal vein tumor recurrence without liver parenchymal invasion following surgical resection. In general, the recurrence sites of colorectal cancer are the lungs and liver, and in the absence of several risk factors for recurrence, curative resection could provide a good long-term prognosis [15]. Otani et al. reported 43 cases of colorectal cancer with adjacent drainage vein tumor thrombosis, and aggressive surgical resection was considered to improve long-term prognosis [11]. In our case, the primary rectal carcinoma itself did not show massive venous (v1) and lymphatic (ly1) invasion; therefore, even after systemic chemotherapy, PVTT could have occurred through this vascular invasion, or CTCs may have been implanted into the portal vein wall.

In the case of HCC, tumor thrombosis is often detected via pathological assessment after surgery, and the presence of portal vein invasion has been reported as a risk factor for recurrence [16, 17]. Surgical removal of the tumor thrombosis was the most effective curative treatment for HCC [18]. However, transcatheter arterial chemoembolization can be considered in patients with severe liver failure or a highly advanced tumor stage [12, 19]. The mechanism of PVTT is different between HCC and colorectal liver metastasis (CRLM). PVTT from HCC is derived from direct invasion, whereas CRLM is considered based on whether direct tumor invasion is through the blood stream or indirect tumor invasion through CTC implantation. The prognosis of patients with venous tumor thrombosis of colorectal cancer is unclear; however, evidence of hepatectomy for CRLM is well established [20]. In HCC, obstructive tumor thrombosis of the bile duct and portal vein thrombosis have been reported, and the dismal prognosis of these conditions could be beneficially changed with curative surgery [21]. Given that metastatic PVTT could be curatively resected, aggressive surgery could potentially be an efficient treatment.

In patients without other distant metastases and with good performance status, aggressive surgical resection should be considered. In our case, early recurrence was noted at the paraaortic lymph nodes, and systemic second-line treatment is currently being administered. Cohen et al. reported that during treatment for metastatic colorectal cancer, the number of CTCs is an independent risk factor for poor overall survival (OS) and progression-free survival. In patients with colorectal metastasis, those with unfavorable CTCs had a dismal prognosis of 3.7 months of OS compared to those with a low number of CTCs with 11.0 months of OS [9]. Even after curative surgery, intrahepatic recurrence occurred approximately 60% [5]. Until now, the relationship between CTC and CRLM remains unclear. Some studies have demonstrated that CTC is associated with long-term survival in various cancer types [22–24]. Given that most metastatic forms of colorectal cancer are liver metastasis, CTCs could be implanted into the portal vein, consequently resulting in PVTT. Early detection of recurrent disease when traditional clinical indicators, such as radiological findings are negative, is important to improve patient survival. Therefore, CTC investigation would be a breakthrough in cancer metastatic mechanism. In our case, the relatively better survival of 15 months following the first surgery could be achieved because of repeat surgical resection combined with systemic chemotherapy.

Radiological findings of tumor thrombosis are quite similar to those of venous thrombosis, but the precise diagnosis is quite difficult with dynamic-enhanced CT alone. Recently, PET yielded good efficacy for detecting venous tumor thrombosis when using intense radiotracer accumulation [25, 26]. Additionally, MRI plays an essential role in differentiating thrombosis and tumor thrombosis, and T2- and diffusion-weighted imaging were shown to be particularly accurate for diagnosis [27]. PET-CT has an important role in diagnosing cancer recurrence and characterizing a thrombus using abnormal accumulation (SUVmax) over time. The mean SUVmax values for bland thrombosis and tumor thrombosis have been shown to be significantly different. For differentiating tumor thrombosis from bland thrombosis, the measurement of SUVmax (cutoff value of 2.25) on PET is useful [26]. In the present case, tumor marker levels remained elevated during systemic chemotherapy. The diagnosis of tumor thrombosis was made based on a SUVmax value of 5.8 on PET-CT. PET-CT enabled the detection of tumor thrombosis recurrence by revealing an elevated SUVmax.

## Conclusions

The recurrence pattern of only portal vein thrombosis from colorectal cancer is extremely rare; however, attention should be paid to tumor thrombosis as a recurrence pattern of colorectal carcinoma. Moreover, the radiological findings of portal vein thrombosis are quite similar to those of PVTT. The present findings reveal that PET plays an important role in distinguishing PVTT and portal vein thrombosis by evaluating SUV. Furthermore, PET can help guide selection of additional treatment, such as surgical resection with systemic chemotherapy.

## Abbreviations

CRLM: Colorectal liver metastasis
CT: Computed tomography
CTCs: Circulating tumor cells
HCC: Hepatocellular carcinoma
IV: Intravenous infusion
MRI: Magnetic resonance imaging
OS: Overall survival
PET: Positron emission tomography
PVTT: Portal vein tumor thrombosis
SOL: Space-occupying lesion
SUVmax: Maximum standardized uptake value
